# Loss of primary cilia and dopaminergic neuroprotection in pathogenic LRRK2-driven and idiopathic Parkinson’s disease

**DOI:** 10.1073/pnas.2402206121

**Published:** 2024-08-01

**Authors:** Shahzad S. Khan, Ebsy Jaimon, Yu-En Lin, Jonas Nikoloff, Francesca Tonelli, Dario R. Alessi, Suzanne R. Pfeffer

**Affiliations:** ^a^Department of Biochemistry, Stanford University School of Medicine, Stanford, CA 94305-5307; ^b^Aligning Science Across Parkinson’s Collaborative Research Network, Chevy Chase, MD 20815; ^c^Medical Research Council Protein Phosphorylation and Ubiquitylation Unit, University of Dundee, Dundee DD1 5EH, Scotland, United Kingdom

**Keywords:** LRRK2 kinase, Rab GTPase, primary cilia, Parkinson’s disease, Hedgehog signaling

## Abstract

Activating leucine-rich repeat kinase 2 (LRRK2) mutations cause Parkinson’s and phosphorylation of Rab10 by pathogenic LRRK2 blocks primary ciliogenesis in cultured cells. In the mouse brain, LRRK2 blockade of primary cilia is highly cell type specific: For example, cholinergic interneurons and astrocytes but not medium spiny neurons of the dorsal striatum lose primary cilia in LRRK2-pathway mutant mice. We show here that the cell type specificity of LRRK2-mediated cilia loss is also seen in human postmortem striatum from patients with LRRK2 pathway mutations and idiopathic Parkinson’s. Single nucleus RNA sequencing shows that cilia loss in mouse cholinergic interneurons is accompanied by decreased glial-derived neurotrophic factor transcription, decreasing neuroprotection for dopamine neurons. Nevertheless, LRRK2 expression differences cannot explain the unique vulnerability of cholinergic neurons to LRRK2 kinase as much higher LRRK2 expression is seen in medium spiny neurons that have normal cilia. In parallel with decreased striatal dopaminergic neurite density, LRRK2 G2019S neurons show increased autism-linked CNTN5 adhesion protein expression; glial cells show significant loss of ferritin heavy chain. These data strongly suggest that loss of cilia in specific striatal cell types decreases neuroprotection for dopamine neurons in mice and human Parkinson’s.

## Significance

Activating mutations in leucine-rich repeat kinase 2 (LRRK2) cause Parkinson’s and block primary cilia formation in specific cells in the mouse brain. Here, we report the same cell-type-specific cilia loss in postmortem brain from Parkinson’s patients with or without LRRK2 pathway mutations. Single-nucleus RNA sequencing of mutant mouse dorsal striatum revealed decreased production of neuroprotective glial-derived neurotrophic factor (GDNF) RNA by cholinergic neurons that sustains dopamine neurons. These experiments highlight the importance of ciliary signaling between dopamine neurons and targets in the striatum with important consequences for dopamine neuron survival.

Mutations in the gene encoding leucine-rich repeat kinase 2 (LRRK2) cause inherited Parkinson’s disease (PD), a neurodegenerative disorder that results in loss of dopaminergic neurons in the Substantia nigra (1–4). LRRK2 has been shown to phosphorylate a subset of Rab GTPases (2, 3), and reversal of LRRK2 phosphorylation is mediated in part by the PPM1H phosphatase (5). LRRK2 action blocks the process of primary cilia formation (6, 7) and PPM1H knockout in wild-type (WT) mouse embryonic fibroblast cells phenocopies the loss of cilia seen upon pathogenic LRRK2 expression (5).

Rab GTPases are master regulators of protein trafficking and carry out their roles by binding to specific, cognate partner proteins when GTP-bound (8, 9). Phosphorylation of Rabs interferes with GTP loading by guanine nucleotide exchange factors, a prerequisite for their binding to effector proteins (2, 3). Instead, once phosphorylated, Rab GTPases bind new sets of phospho-specific protein effectors. For Rab8 and Rab10, these include RILPL1, RILPL2, JIP3, JIP4, and Myosin Va proteins (3, 6, 10, 11). These phospho–Rab interactions block ciliogenesis in cell culture and mouse brain via a process that requires RILPL1 and Rab10 proteins (3, 6, 7); centriolar cohesion is also altered (12, 13).

The dorsal striatum is composed of medium spiny neurons, interneurons, and glial cells such as astrocytes, and this region is infiltrated by the extensive processes of dopamine-secreting neurons from the Substantia nigra (14). Nigral dopaminergic neurons secrete Sonic Hedgehog (Shh) that is sensed by poorly abundant, striatal cholinergic interneurons (15). Shh is needed for the survival of both the cholinergic target cells and the Shh-producing dopamine neurons, even though only the cholinergic neurons express the PTCH1 Shh receptor. Shh triggers the secretion of glial-derived neurotrophic factor (GDNF) from the cholinergic neurons, which provides reciprocal neuroprotection for the dopaminergic neurons of the Substantia nigra (15).

We showed previously that rare, striatal, cholinergic interneurons that would normally sense Shh via their primary cilia are significantly less ciliated in four different mouse models of pathogenic LRRK2 disease [LRRK2 G2019S knock in or transgene, R1441C knock in, or PPM1H knockout (6, 16)]. Remarkably, we report analogous, cell-type-specific cilia loss in human postmortem striatum from patients with both idiopathic and LRRK2-pathway-based PD.

In prior work, we predicted that cilia loss would decrease the ability of cells to sense Shh signals since Shh signaling is primary cilia-dependent; we find strong evidence of Shh signaling dysfunction due to cilia loss in cholinergic neurons as well as astrocytes that share a broad ciliary deficit. Here, we also report single nucleus RNA sequencing of the dorsal striatum of WT and G2019S LRRK2 mutant mice. We see decreased GDNF transcripts in cilia-deficient LRRK2 G2019S cholinergic neurons, increases in the RNA encoding the autism-spectrum disorder-associated Contactin 5 neuronal adhesion protein, and down regulation of the iron-binding ferritin heavy chain mRNA. This work provides fundamental information regarding the consequences of LRRK2 mutation and the molecular basis of PD.

## Results

**Fig. 1.**
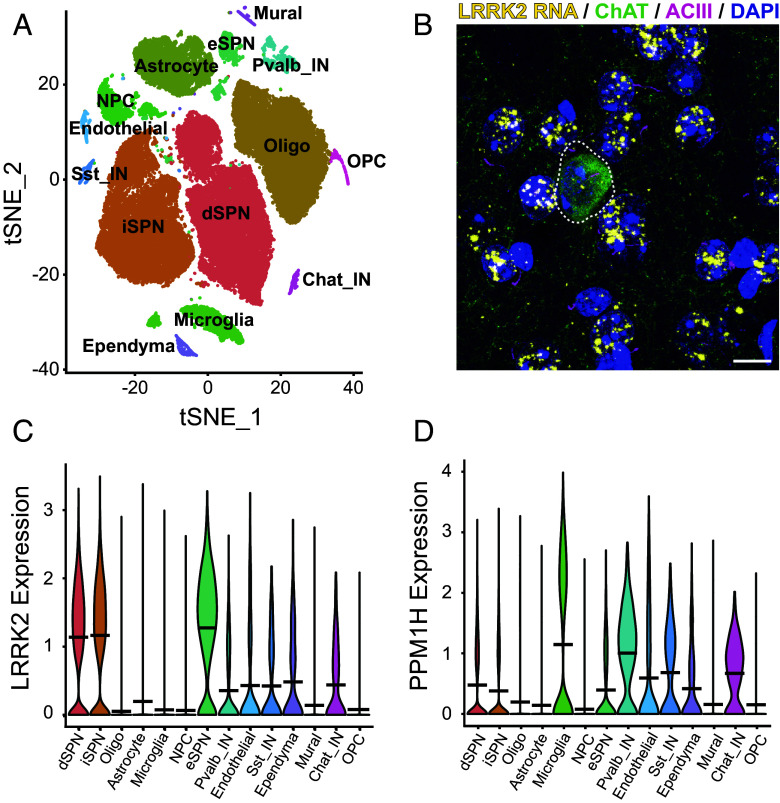
Single nucleus RNA sequencing analysis of WT mouse dorsal striatum. (*A*) tSNE plot showing cell types detected using markers summarized in *SI Appendix*, Table S1. (*B*) RNAScope in situ hybridization to detect LRRK2 transcripts in the dorsal striatum. Green, anti-choline acetyltransferase staining of a cholinergic interneuron surrounded by the much more abundant medium spiny neurons; pink, primary cilia; yellow dots, LRRK2 transcripts, blue, DAPI stained nuclei. Bar, 10 µm. (*C* and *D*) Comparison of relative LRRK2 (*C*) or PPM1H (*D*) RNA levels in cell types color coded as in *A*.

In an effort to determine why cilia are only lost in certain cell types in the striatum of mice harboring activating LRRK2 mutations or missing the PPM1H phosphatase, we carried out single-nucleus RNA sequencing (snRNAseq) of dorsal striatum from 6-mo-old, WT and LRRK2 G2019S mice. Such an analysis enables us to examine cell-type-specific gene expression changes due to LRRK2 G2019S expression. Shown in Fig. 1*A* is a t-distributed Stochastic Neighbor Embedding (tSNE) plot used to identify distinct cell types in this brain region of WT mice. These clusters represent 54,023 nuclei that were sequenced from 6 female mice. As expected, the largest clusters represent the direct and indirect spiny neurons that express D1 and D2 dopamine receptors, respectively, followed by oligodendrocytes, astrocytes, and microglia. Much smaller clusters were detected representing neuronal precursor cells, eccentric spiny neurons, ependymal, endothelial, and mural cells, as well as parvalbumin, cholinergic, and somatostatin-expressing interneurons (Fig. 1*A*). Clusters were identified using established markers of each cell type in this brain region (*SI Appendix*, Table S1) (17–22); clustering was based on 14 cell types in this representation. The top ten genes in the cholinergic interneuron and astrocyte subclusters are presented in *SI Appendix*, Table S2; the entire dataset can be found in Dataset S1.

One reason why astrocytes and cholinergic neurons but not the much more abundant medium spiny neurons lose their cilia might be due to their relative content of LRRK2 kinase or the counteracting, PPM1H phosphatase. SnRNAseq showed that at least at the RNA level, the reverse is true: Direct comparison of RNA transcript abundance showed that direct, indirect, and eccentric spiny neurons express the highest relative amounts of LRRK2 RNA compared with astrocytes and cholinergic interneurons (Fig. 1*C*). Microglia, parvalbumin, and cholinergic interneurons also expressed slightly higher levels of PPM1H than other cell types (Fig. 1*D*). RNAscope fluorescence in situ hybridization confirmed this discrepancy as shown in Fig. 1*B*; the predominant, ciliated medium spiny neurons showed many more LRRK2 transcript dots (abundant yellow spots on blue nuclei) than any of the cholinergic interneurons labeled in green with anti-choline acetyltransferase antibody (circled with a dashed line, Fig. 1*B*; cilia on all neurons are shown in pink). Thus, cell-type-specific, high LRRK2 levels do not necessarily predict cilia loss in the striatum.

### Two Classes of Dorsal Striatal Cholinergic Interneurons.

**Fig. 2.**
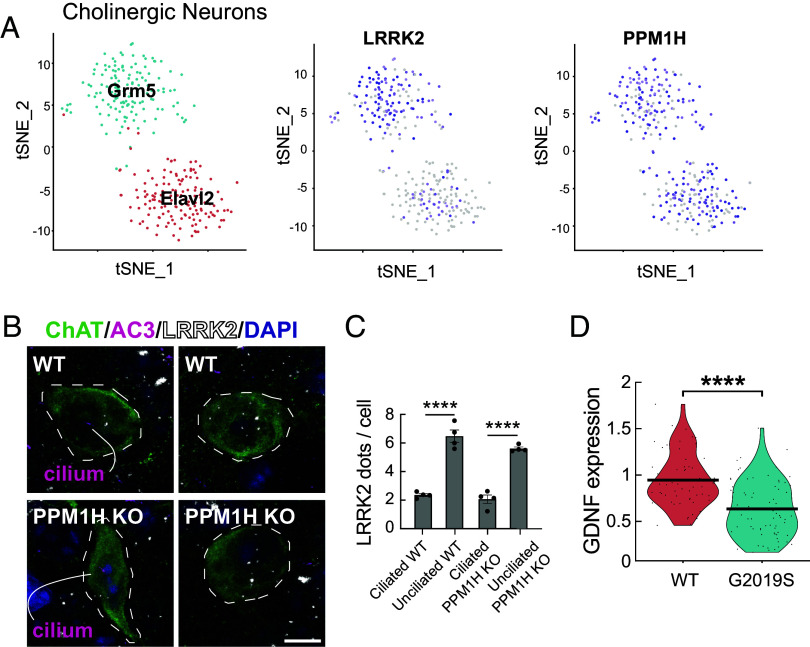
Two classes of cells explain ciliation differences in cholinergic interneurons in the dorsal striatum. (*A*) Single nucleus RNA sequencing analysis of cholinergic interneurons from WT mouse dorsal striatum. Cholinergic interneurons subclustered according to the markers indicated. Relative LRRK2 and PPM1H expression is shown at the *Right* for all subclusters with purple intensity reflecting abundance in that nucleus. (*B* and *C*) RNAScope in situ hybridization analysis of LRRK2 RNA (white dots in *B*) in cholinergic interneurons according to ciliation status in 2.5-mo-old WT or PPM1H KO dorsal striatum. Error bars in *C* represent SEM from 4 WT and 4 PPM1H KO brains, with >25 ChAT^+^ neurons scored per brain. Statistical significance was determined using an unpaired *t* test: ****P < 0.0001 for ciliated WT versus unciliated WT; ****P < 0.0001 for ciliated PPM1H KO versus unciliated PPM1H KO. (*D*) Comparison of GDNF expression from RNAseq data between WT and G2019S KI cholinergic interneurons. Statistical significance was determined using a student’s *t* test. ****P < 0.0001.

Two classes of striatal cholinergic interneurons were identified based on expression of ELAV Like RNA Binding Protein 2 (*Elavl2*) and Glutamate Metabotropic Receptor 5 (*Grm5*) (Fig. 2 *A*, *Left*). The *Elavl2* cluster showed lower LRRK2 expression compared with the Grm5 cluster (Fig. 2 *A*, *Middle*); both clusters expressed comparable levels of PPM1H phosphatase (Fig. 2 *A*, *Right*). As reviewed by Ahmed et al. (23), subsets of cholinergic interneurons express either Zic4 and originate from the Septal Epithelium or Lhx6 and originate from the Medial Ganglionic Eminence; Gbx2 is expressed in almost all cholinergic interneurons. Zic4 and Lhx6 were expressed at comparable levels between the two classes of cholinergic neurons we captured; by contrast, we did not detect Gbx2 expression. From this, we conclude preliminarily that the two classes are of mixed developmental origin.

Because only about half of cholinergic interneurons lose their cilia in the G2019S LRRK2 dorsal striatum (6), we were curious to determine whether the cells that lose cilia correspond to the Grm5 subcluster that expresses higher LRRK2. Quantification of ciliation and LRRK2 RNA levels using RNAscope in situ hybridization on dorsal striatal sections revealed that in both WT and PPM1H knockout mice that phenocopy LRRK2 mutations and have fewer cilia (16), unciliated cholinergic interneurons did indeed express higher levels of LRRK2 compared with ciliated cells (Fig. 2 *B*, white dots, and *C*). Thus, in cells such as cholinergic interneurons that are sensitive to LRRK2 inhibition of ciliogenesis, LRRK2 expression levels directly correlate with cilia loss. Note that WT cells are ~70% ciliated compared with ~35% ciliation in PPM1H knockout striatum (16).

**Fig. 3.**
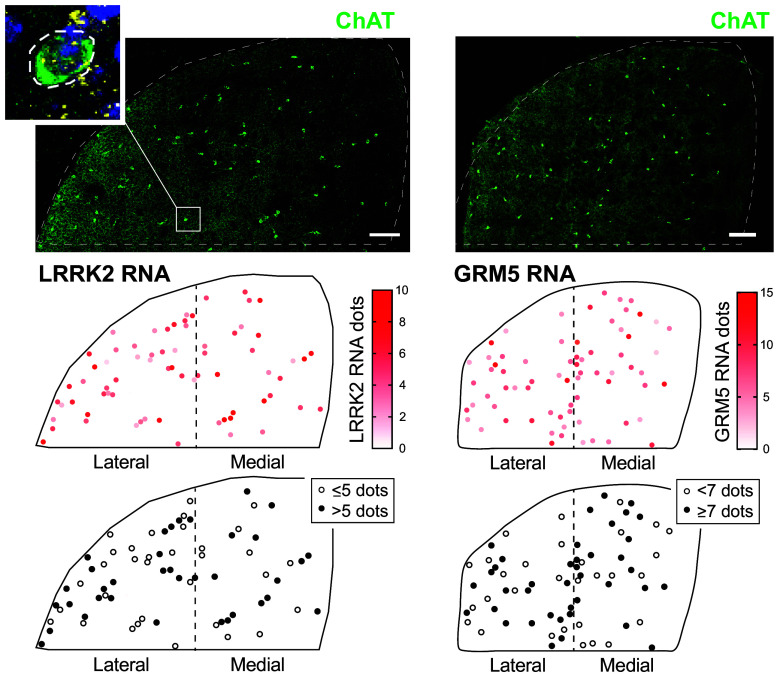
Spatial distribution of LRRK2 (*Left* column) and GRM5 RNA (*Right* column) levels in mouse dorsal striatal cholinergic interneurons. *Top* row: representative tile scan images of mouse dorsal striatum from 2.5-mo-old WT mice. Cholinergic interneurons were labeled using anti-ChAT antibody (green) and LRRK2 RNA or GRM5 RNA (yellow dots) were detected using RNAScope in situ hybridization. Nuclei were labeled using DAPI (blue). *Inset* at *Upper Left* shows an example of an enlarged cholinergic interneuron and its LRRK2 RNA content (yellow dots) used to quantify expression summarized in lower rows. *Middle* row: Spatial heat maps of LRRK2 RNA (*Left*) and GRM5 RNA (*Right*) in cholinergic interneurons. Each dot represents one cholinergic interneuron color coded based on RNA expression. Red intensity is used to indicate number of RNAScope dots in each cell. *Bottom* row: Same as in *Middle* row except that Black dots represent cholinergic interneurons with high LRRK2 (>5 dots per cell) or high GRM5 (≥7 dots per cell) and white dots represent cholinergic interneurons with low LRRK2 (≤5 dots per cell) or low GRM5 (<7 dots per cell). (Bar, 200 µm.)

Fig. 3 shows the distribution of cholinergic interneurons across the dorsal striatum and their unique, cell-by-cell expression of LRRK2 RNA (*Left* column) or GRM5 RNA (*Right* column). Cells were labeled for the indicated RNAs using RNAScope (as in Fig. 2*B*) and quantified manually at high magnification as summarized in the heat map and summary diagrams. These data show that high and low LRRK2 or GRM5 expressing cholinergic neurons are randomly distributed across the dorsolateral and dorsomedial striatum; those expressing higher levels of LRRK2 will be most vulnerable to cilia loss (Fig. 2*C*).

### Effect of LRRK2 Mutation on Dorsal Striatal Gene Expression.

Previous work suggested that Shh signaling triggers GDNF production by striatal cholinergic interneurons (15). Since Hedgehog signaling is primary cilia-dependent (24, 25), we reasoned that loss of cilia due to LRRK2 mutation would decrease Hedgehog-responsive gene expression, including that of GDNF. Fig. 2*D* compares the levels of GDNF RNA specifically in cholinergic neurons of the dorsal striatum of WT and LRRK2 G2019S mice (from snRNAseq analysis); these neurons showed a highly significant decrease in GDNF transcription, as would be predicted from earlier findings (15). These data demonstrate that LRRK2 mutant mice are defective in providing critical neuroprotective factors for dopamine neurons.

**Fig. 4.**
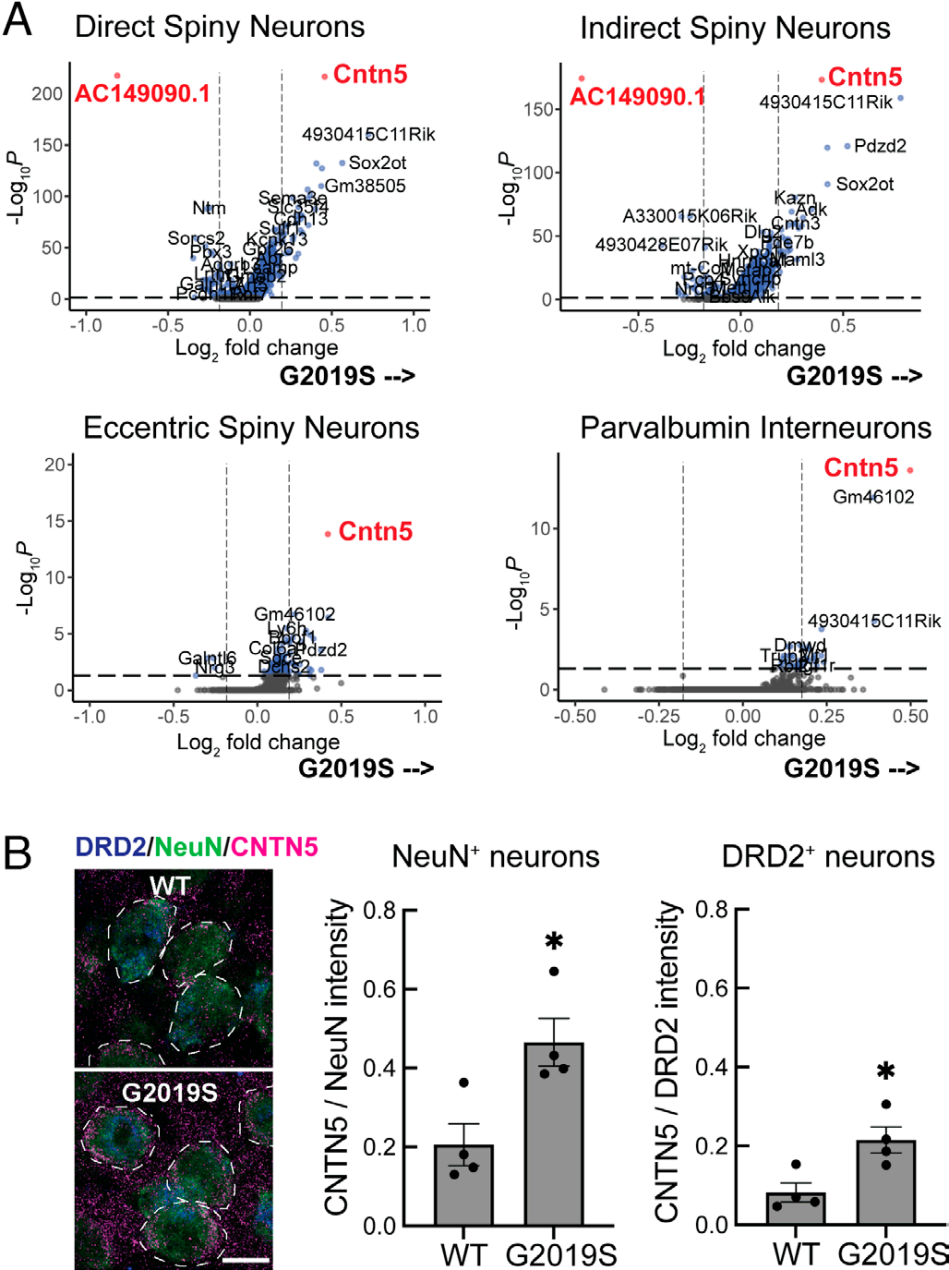
(*A*) Volcano plot analysis comparing transcripts that increase (*Right* side) or decrease (*Left* side) in LRRK2 G2019S dorsal striatal neurons compared with age-matched WT mouse controls. Dashed vertical lines represent the Log2 fold change cutoff (between −0.2 and 0.2). The dashed horizontal line represents the adjusted *P* value cutoff (less than 0.05). The cell type analyzed is indicated at the *Top* of each graph. Number of variables for direct spiny projection neurons (7,699), indirect spiny projection neurons (7,213), eccentric spiny projection neurons (8,212), and parvalbumin interneurons (9,270). (*B*) Immunofluorescence microscopy of cells stained with antibodies to detect NeuN (green), DRD2 (blue), and CNTN5 (red) expression. Total fluorescence intensity of CNTN5 within 5 pixels of the NeuN or DRD2 was quantified and normalized to either total NeuN staining or total DRD2 staining. Error bars represent SEM from 4 WT and 4 G2019S brains, with >365 NeuN^+^ neurons and >200 DRD2^+^ neurons scored per brain. Statistical significance was determined using an unpaired *t* test. CNTN5 intensity in NeuN^+^ neurons: *P = 0.0184 for WT versus G2019S and CNTN5 intensity in DRD2^+^ neurons: *P = 0.0177 for WT versus G2019S. (Bar, 10 µm.) Detailed precise data can be found in Dataset S1.

To further evaluate cellular changes in additional cell types associated with the LRRK2 G2019S mutation, significant differences in RNA levels were compared for each cell type (Fig. 4 and Dataset S1). All transcripts shown on the *Right* side of each of the volcano plots increased in G2019S animals; transcripts shown on the *Left* side decreased in G2019S compared with WT mice. Fig. 4 compares changes seen in direct, indirect, and eccentric spiny neurons as well as Parvalbumin interneurons. The first overall conclusion from these experiments is that gene expression changes are rather minor; AC149090.1, an ortholog of Phosphatidylserine Decarboxylase, was decreased in eight of the G2019S LRRK2 clusters we assessed (Chat_IN, Parvalbumin_IN, dSPN, iSPN, eSPN, Oligos, All Astrocytes, and Crym_Astrocytes). For direct medium spiny neurons, relatively few genes were down-regulated in G2019S LRRK2 animals; most changes were increases in gene expression. Nevertheless, especially noteworthy was the common increase in the RNA encoding Contactin-5 (CNTN5), an Ig-domain containing neural cell adhesion protein [also known as “NB-3” (26) linked to attention-deficit/hyperactivity disorder and autism spectrum disorder (27–29)]. CNTN5 interacts with CNTNAP4 as a scaffold on interneurons to support the growth of ganglion cells (30–32); CNTN5^−/+^ iPSC-derived glutamatergic neurons show intensified excitatory neuron synaptic activity in culture (33).

Anti-CNTN5 antibody staining confirmed at least a twofold increase in CNTN5 protein in LRRK2 G2019S NeuN-expressing neurons and DRD2-expressing indirect spiny neurons (Fig. 4*B*). Our working hypothesis is that loss of dopaminergic processes in the striatum of G2019S LRRK2 mice leads to upregulation of CNTN5 in spiny neurons as an attempt to strengthen or retain nigral-striatal cell–cell interactions.

### Loss of Dopamine Projections and GDNF Receptor Expression in Mouse.

**Fig. 5.**
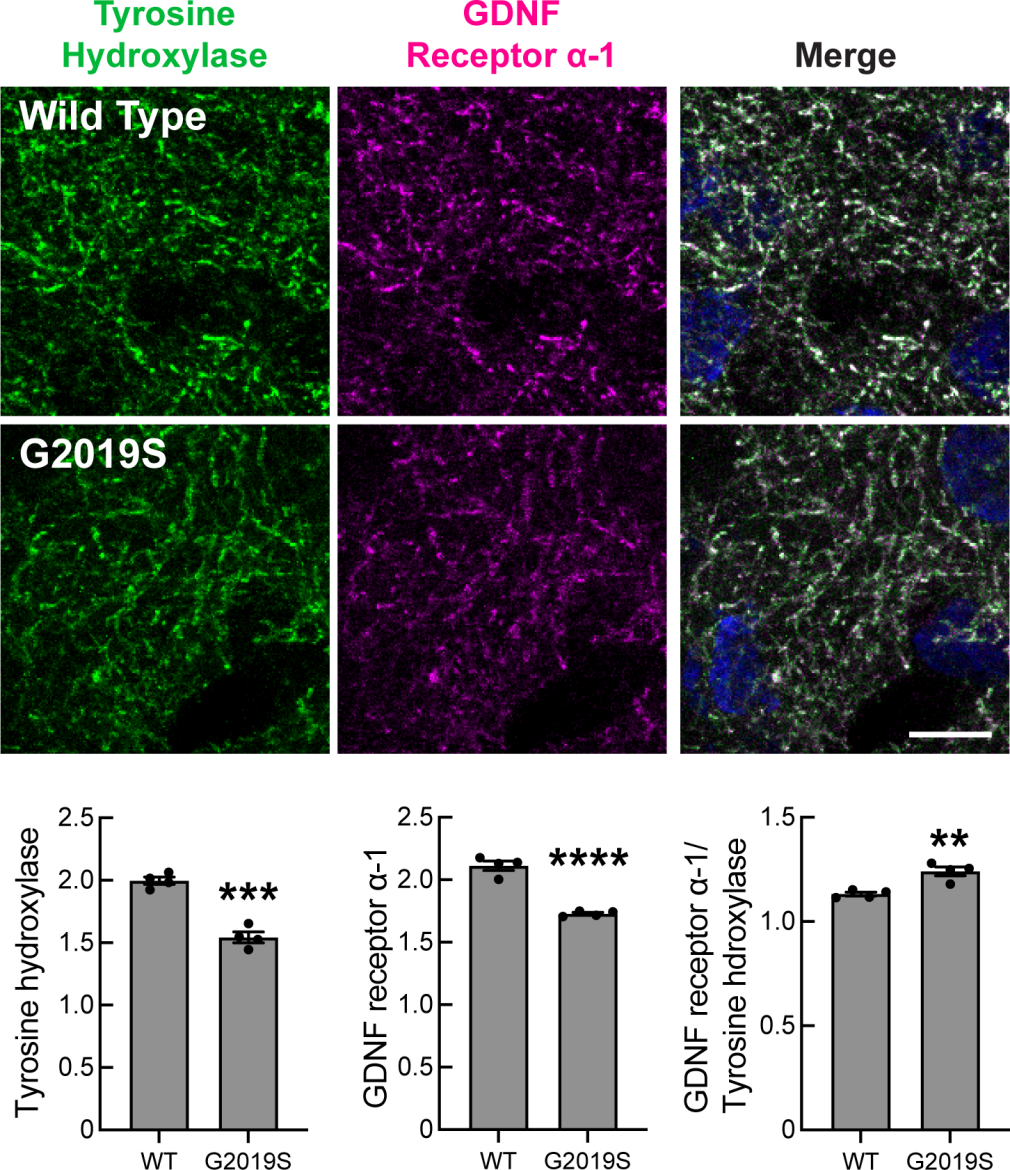
Loss of tyrosine hydroxylase and GDNF receptor staining in mouse G2019S LRRK2 dorsal striatum. *Top* panels: Confocal images of tissue labeled with anti-Tyrosine hydroxylase antibodies (green) and anti-GDNF receptor alpha-1 antibodies (magenta) in sections of the dorsal striatum from 5-mo-old WT or G2019S LRRK2 KI mice; blue stain in the merge image, NeuN. Unidentified tissue samples were stained and imaged at the same time; images were normalized by NeuN staining intensity but required very little normalization. *Bottom* graphs: Integrated intensity of Tyrosine hydroxylase and GDNF receptor α-1 was quantified using CellProfiler. Error bars represent SEM from 4 WT and 4 G2019S brains, with >25 fields scored per brain. Statistical significance was determined using an unpaired *t* test. Intensity of Tyrosine hydroxylase: ***P = 0.0001 for WT versus G2019S, Intensity of GDNF receptor α-1: ****P < 0.0001 for WT versus G2019S and intensity of GDNF receptor α-1/Tyrosine Hydroxylase: **P = 0.0034 for WT versus G2019S. (Bar, 10 µm.)

In mice engineered such that Shh is not expressed by dopaminergic neurons, Gonzalez-Reyes et al. (15) reported a progressive reduction in striatal GDNF mRNA and protein, and at 12 mo, upregulation of the Ret GDNF receptor and its coreceptor GDNF Receptor Alpha 1, which binds all members of the GDNF ligand family. In 5-mo-old G2019S LRRK2 mice, we observed a significant ~23% loss of striatal tyrosine hydroxylase staining compared with WT mice, using direct quantitation of fluorescent antibody staining (Fig. 5), consistent with previous reports of dopaminergic neurite loss (34–37). At this stage, the level of the GFRA1 GDNF receptor present on the dopaminergic processes decreased to the about same extent as tyrosine hydroxylase; only very slight upregulation was detected (Fig. 5, *Lower Right* panel). The loss of striatal dopaminergic projections in G2019S LRRK2 animals is consistent with the upregulation of CNTN5 observed in the striatal spiny neurons (Fig. 4), in accord with our working hypothesis.

### Four Classes of Dorsal Striatal Astrocytes.

**Fig. 6.**
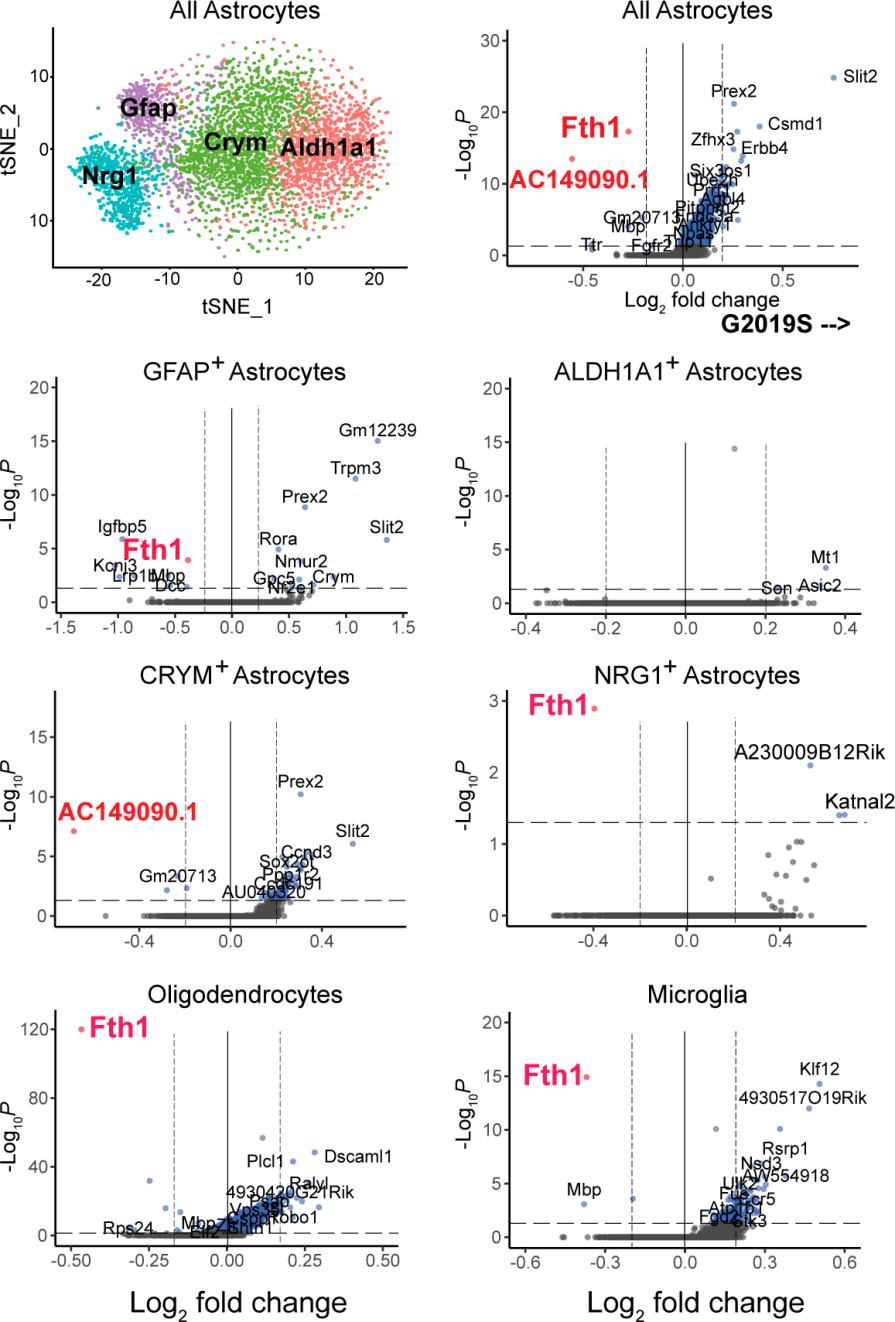
Volcano plot analysis comparing transcripts that increase (*Right* side) or decrease (*Left* side) in LRRK2 G2019S dorsal striatal astrocytes, oligodendrocytes, or microglia compared with age-matched WT mouse controls. Dashed vertical lines represent the Log2 fold change cutoff (between −0.2 and 0.2). The dashed horizontal line represents the adjusted *P* value cutoff (less than 0.05). *Upper Left*, t-SNE representation of single nucleus RNA sequencing of astrocytes from WT mouse dorsal striatum, subclustered according to the markers indicated. Each cell type analyzed is indicated at the *Top* of each volcano plot. Number of genes for total astrocytes (4,791), GFAP+ astrocytes (4,463), ALDH1A1+ astrocytes (4,901), CRYM+ astrocytes (4,027), NRG1+ astrocytes (4,011), Oligodendrocytes (3,522 genes), and Microglia (3,606). Detailed precise data can be found in Dataset S1.

tSNE cluster analysis of astrocytes in the dorsal striatum revealed four classes of cell types based on their relative levels of Aldehyde Dehydrogenase 1A1 (*Aldh1a1*), µ-crystallin (*Crym*), Neuregulin 1 *(Nrg1*), and GFAP (*Gfap*) RNAs (Fig. 6, *Upper Left*). Chai et al. (38) noted previously that μ-crystallin displays a gradient of expression in the striatum, consistent with our findings. As reviewed by Khakh (39), GFAP is generally a poor marker of striatal astrocytes, while antibodies against Aldh1l1, S100β, GLT1, and Kir4.1 label most striatal astrocytes. By contrast, the astrocyte GABA transporter GAT-3 labels ~30% of evenly distributed astrocytes. Furthermore, μ-crystallin labels ~85% of the astrocytes in the ventral striatum, but only ~30% in the dorsal regions, even though the density of astrocytes is equivalent. Our sequencing data are consistent with these observations in that the four astrocyte classes showed equal expression of Aldh1l1, S100β, GLT, and Kir4.1, and included both GAT-3-positive and GAT-3 negative nuclei. In addition, the four astrocyte subtypes showed comparable levels of LRRK2 and PPM1H; we do not yet know whether any subcluster is more vulnerable to cilia loss than another.

### Ferritin Heavy Chain Dysregulation in LRRK2 Mutant Glial Cells.

Fig. 6 shows gene expression changes seen in LRRK2 G2019S dorsal striatum compared with WT mice for all astrocytes, oligodendrocytes, and microglia and astrocytes segregated according to individual clusters (as in Fig. 2). This comparison revealed that oligodendrocytes, astrocytes, and microglia all showed a decrease in Ferritin heavy chain (Fth1) transcription in G2019S LRRK2 dorsal striatum (Fig. 6). Fth1 sequesters iron within the ferritin complex, reducing the availability of free iron that can lead to the generation of reactive oxygen species and lipid peroxides (40). A decrease in Fth1 will disrupt iron homeostasis and antioxidant defenses, increasing the likelihood of ferroptosis. Transcription of the FTH1 gene is closely tied to cellular iron levels and is normally repressed under conditions of low iron (41). Additional work will be needed to understand why FTH1 RNA decreases in LRRK2 G2019S oligodendrocytes, astrocytes, and microglia, and whether these cells are indeed more vulnerable to ferroptosis. Low brain ferritin has been previously reported in the Substantia nigra, caudate putamen, globus pallidus, cerebral cortex, and cerebellum in human PD (42, 43), and mouse brains deficient in ferritin heavy chain show evidence of oxidative stress (44).

### Ciliogenesis Defects in Human PD Brain.

**Fig. 7.**
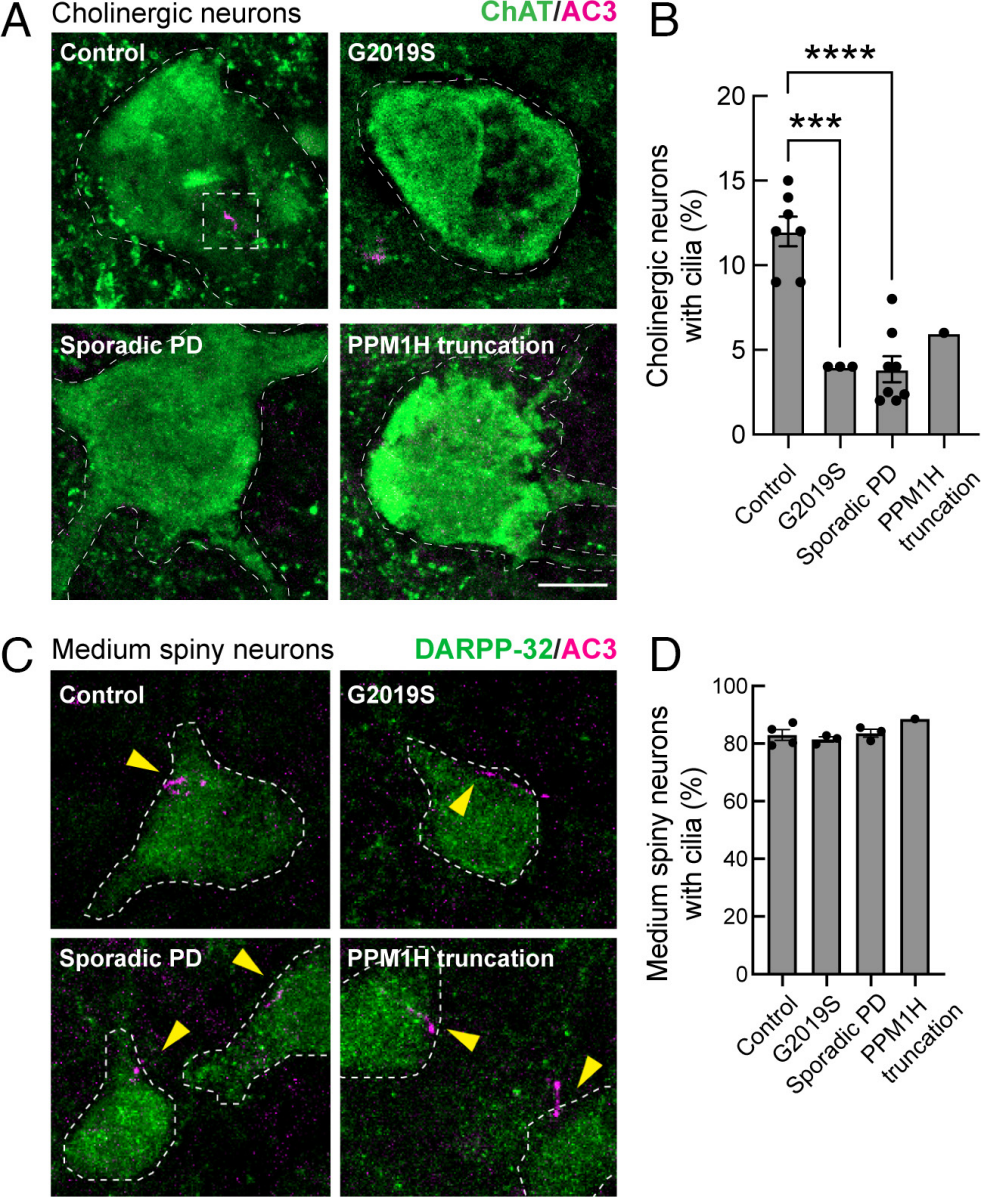
Loss of primary cilia in human cholinergic neurons but not medium spiny neurons from postmortem lenticular nucleus brain sections from PD patients and age-matched controls. (*A*) Confocal microscopy of tissue labeled with anti-ChAT antibody (green) and anti-AC3 antibody (magenta) to identify primary cilia. (*B*) Quantitation of the percentage of ChAT^+^ neurons containing a cilium. Error bars represent SEM from 7 Control, 3 G2019S, 8 sporadic PD, and 1 PPM1H mutation brains, with >30 ChAT^+^ neurons scored. Statistical significance was determined using one-way ANOVA. ***P = 0.0002 for Control versus G2019S PD and ****P < 0.0001 for Control versus Sporadic PD. (Bar, 10 µm.) (*C*) Confocal microscopy of human lenticular nucleus tissue labeled with anti-DARPP-32 antibody (green) to label medium spiny neurons and anti-AC3 antibody (magenta) to identify primary cilia. (*D*) Quantitation of the percentage of DARPP32^+^ neurons containing a cilium. Error bars represent SEM from 4 Control, 3 G2019S, 3 sporadic PD, and 1 PPM1H mutation brains, with >60 DARPP32^+^ neurons scored. All samples were scored blind and evaluated by at least two independent scientists. (Bar, 10 µm.)

Given the importance of primary cilia in Shh signaling and the cell-type selective cilia loss observed in mouse brain, we investigated whether similar cilia loss is detected in human tissue. *SI Appendix*, Table S1 summarizes the health status of the patients from which the postmortem tissue samples were obtained (45). As we have reported previously for mouse striatum, human lenticular nucleus astrocytes and cholinergic interneurons also lose cilia in patients with LRRK2 pathway mutations as well as in patients with idiopathic PD. Fig. 7*A* shows examples of adenylate cyclase 3 (AC3)-labeled cilia (pink) in control, human cholinergic neurons, identified using anti-choline acetyltransferase (ChAT) antibodies (green). Also shown are cholinergic neurons and their lack of cilia in lenticular nucleus sections from human G2019S LRRK2 carriers, patients with sporadic PD, and a rare patient with a detrimental truncation in the PPM1H phosphatase that counteracts LRRK2 action. In mice, cilia elongate from postnatal day 7, stabilizing between P30 and P60, with ~70% ciliation and no cilia loss up to 1 y of age. The control human samples from patients that were ~85 y of age were only 12% ciliated, consistent with age-related cilia loss at later ages in humans (Fig. 7*B*). In contrast, all of the PD samples showed a major loss of primary cilia (Fig. 7 *A* and *B*). Without cilia, these cells will not be able to sense Shh and respond by producing neurotrophic factors. Also noteworthy was our confirmation that medium spiny neurons in the human lenticular nucleus retain their primary cilia, analogous to their counterparts in mouse brain: >80% of medium spiny neurons were ciliated in control and PD patient brain samples (Fig. 7 *C* and *D*).

**Fig. 8.**
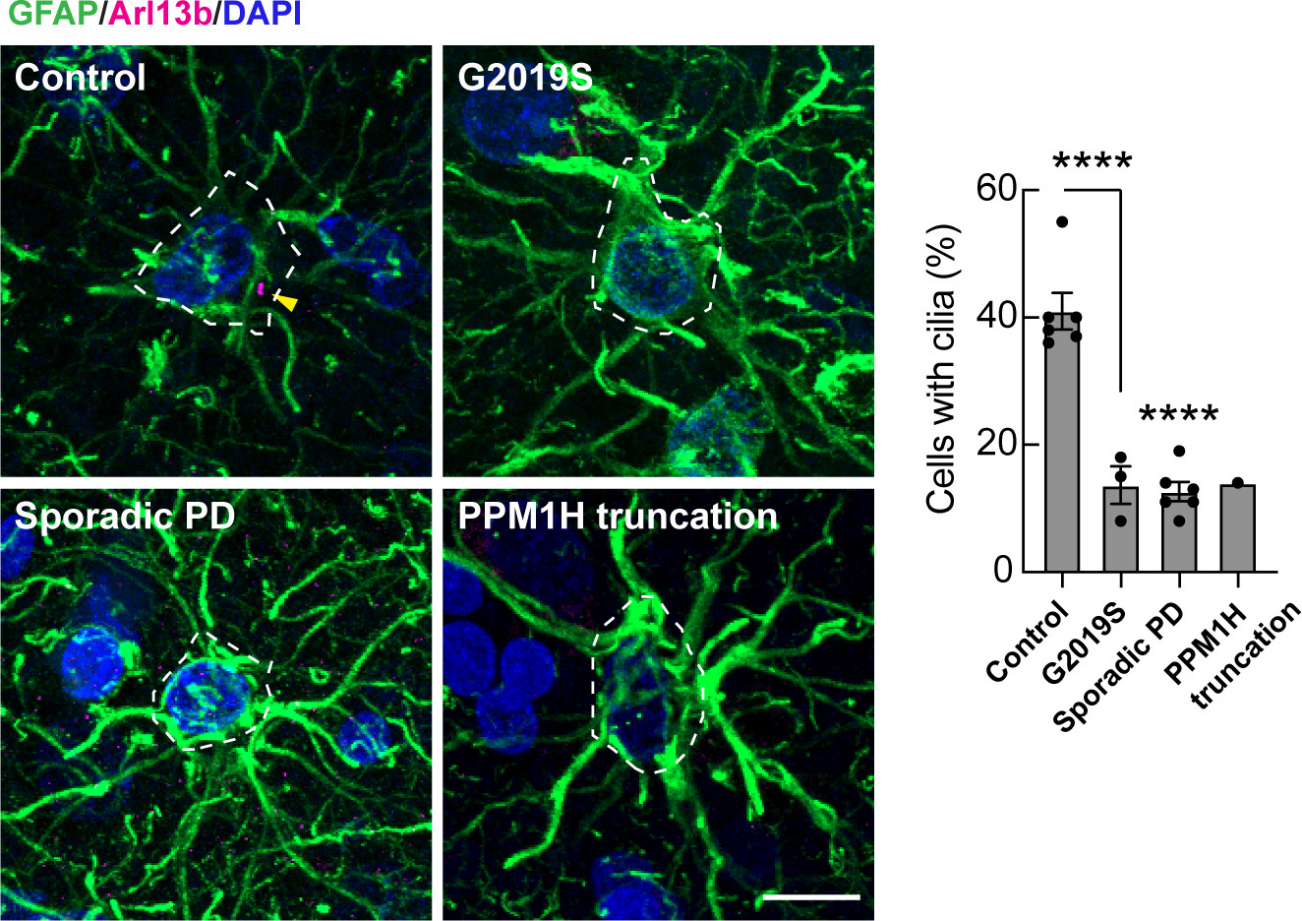
Loss of primary cilia in striatal astrocytes from postmortem brain lenticular nucleus sections from PD patients. *Left* panels. Confocal images of astrocytes labeled using anti-GFAP (green) and primary cilia labeled using anti-Arl13b (magenta). Nuclei were labeled using DAPI (blue). *Right* panels. Quantitation of the percentage of GFAP^+^ astrocytes containing a cilium. Error bars represent SEM from 6 Control, 3 G2019S, 6 sporadic PD, and 1 PPM1H mutation brains, with >30 GFAP^+^ astrocytes scored. All samples were scored blind and evaluated by at least two independent scientists. Statistical significance was determined using one-way ANOVA. ****P < 0.0001 for Control versus G2019S and ****P < 0.0001 for Control versus Sporadic PD. (Bar, 10 µm.)

Analysis of cilia in GFAP^+^ astrocytes using anti-Arl13B antibodies showed similar ciliary loss in G2091S LRRK2 patient carriers, sporadic PD, and in the patient with a PPM1H truncation (Fig. 8). Control GFAP^+^ astrocytes were ~40% ciliated while all PD samples showed 10 to 15% ciliation. These data show that astrocyte cilia are more resistant to loss than cholinergic interneurons due to aging yet are still vulnerable to changes associated with PD.

### Loss of Striatal Cholinergic Interneurons in Mice and Humans.

**Fig. 9.**
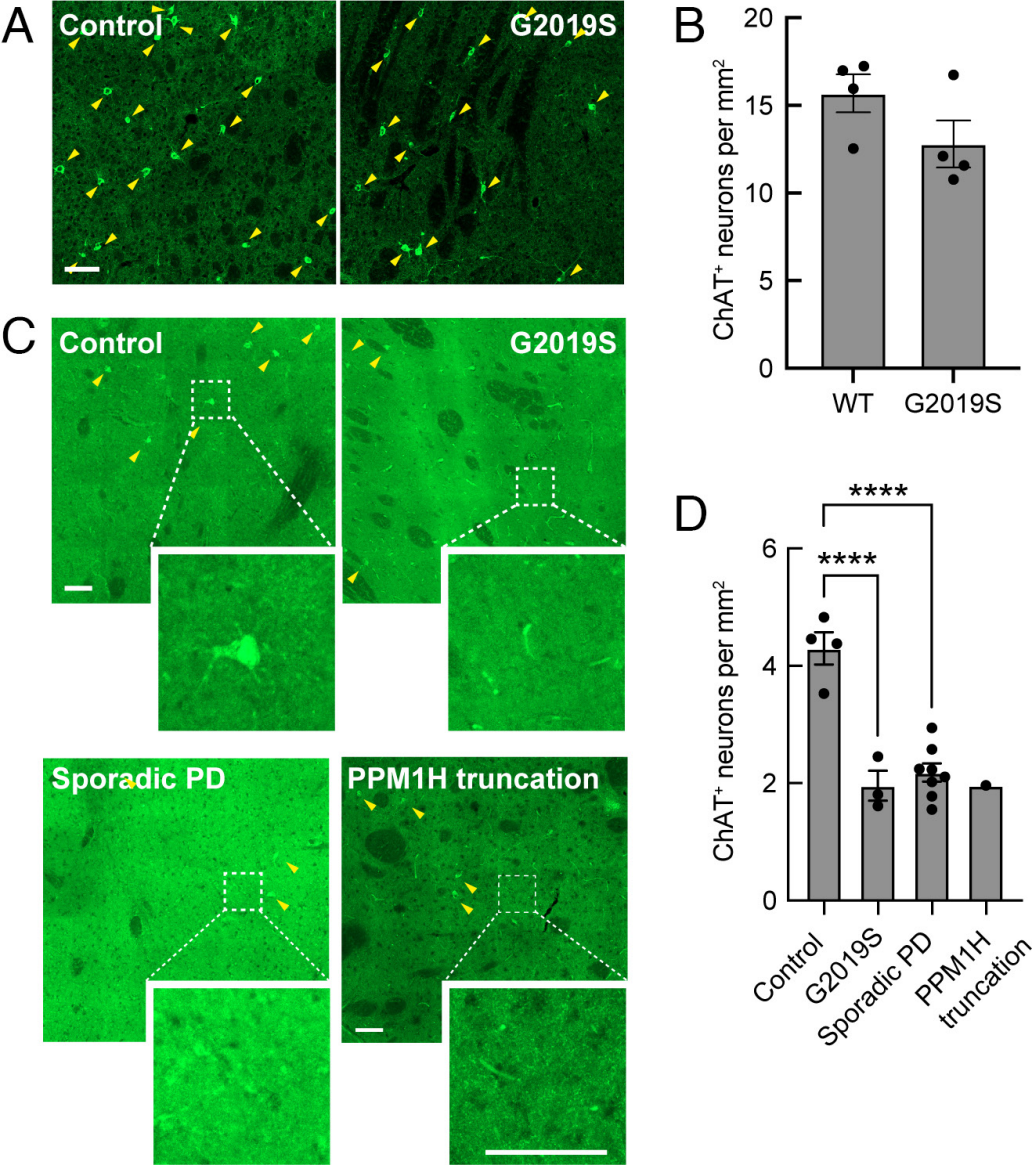
Loss of cholinergic neurons in the dorsal striatum of mice and lenticular nucleus of Parkinson’s patients. (*A*) Representative tile scan images of the mouse dorsal striatum from 5-mo-old WT or G2019S LRRK2 KI mice. Cholinergic interneurons were labeled using anti-ChAT (green). (*B*) Quantitation of mouse ChAT^+^ neurons detected per sq mm. Error bars represent SEM from 4 WT and 4 G2019S brains, with 4 sections scored per mouse. (*C*) Representative tile scan images of the lenticular nucleus from postmortem brain sections from PD patients, identified geographically and molecularly using anti-DARPP-32 antibodies; cholinergic interneurons were labeled using anti-ChAT antibodies (green). (*D*) Quantitation of human ChAT^+^ neurons detected per sq mm. Error bars represent SEM from 4 Control, 3 genetic PD, 8 sporadic PD, and 1 PPM1H mutated brains, with >100 tiles scored per brain. Areas boxed with dashed lines are enlarged and shown. Statistical significance was determined using one-way ANOVA. ****P < 0.0001 for Control versus G2019S and ****P < 0.0001 for Control versus Sporadic PD. (Bar, 100 µm.)

In mice lacking Shh production in dopamine neurons, progressive degeneration of cholinergic neurons was observed (15). In the 5-mo-old LRRK2 G2019S mouse striatum, we detected a trend of loss of cell number, from roughly 16 cells per mm^2^ to ~13 cells per mm^2^ (Fig. 9 *A* and *B*). However, in PD patient-derived brain tissue from G2019S carriers or individuals with idiopathic PD (8 brains analyzed), we noted a roughly twofold loss of cholinergic cell bodies over millimeter regions of the striatum (Fig. 9 *C* and *D*). This is consistent with cilia loss that will decrease the ability of the interneurons to receive trophic signals including Shh from dopamine neurons or nearby Parvalbumin interneurons that also produce Shh. Because of the importance of cholinergic interneurons in coordinating the activity of striatal circuits (46), cell loss will also have important consequences for PD patients.

## Discussion

We have shown here that pathogenic LRRK2-driven cilia loss in cholinergic interneurons of the mouse dorsal striatum leads to a decrease in Shh-dependent GDNF transcription in these cells that is proportional to the extent with which they lose their cilia. Because cilia are needed for Shh signal transduction, these findings align with prior work that showed that loss of Shh expression in dopamine neurons decreases GDNF production overall in the striatum and leads to death of both cholinergic and dopaminergic neurons (15). We detect loss of tyrosine hydroxylase-positive dopaminergic processes in the striatum in 5-mo-old G2019S LRRK2 mice, consistent with decreased availability of GDNF as a dopaminergic trophic factor; no upregulation of GDNF receptors was detected at this stage.

Prior reports have come to different conclusions as to whether LRRK2 mutant mice show dopaminergic neuron loss (34–37; see also refs. 47–52). Note that the experiments presented here were carried out using fluorescently tagged antibodies and at high magnification and focus on striatal processes rather than cell bodies in the Substantia nigra. Also, many prior studies used nonlinear, HRP amplification methods and lower magnification and may have missed more subtle decreases in tyrosine hydroxylase levels in fine processes that extend into the dorsal striatum. Under these conditions, we found that the medium spiny neurons upregulate CNTN5 transcription, perhaps as part of a last-ditch attempt to retain or stabilize interneuronal synaptic contacts.

Our single nucleus RNA sequencing revealed two classes of striatal cholinergic interneurons based on expression of *Elavl2* and *Grm5*. Interestingly, the *Elavl2* cluster showed lower LRRK2 expression compared with the *Grm5* cluster. ELAVL2, also called HuB, is an RNA binding protein that binds U-rich motifs in mRNAs in the 3′UTR, 5′UTR, and introns of mRNAs and controls gene expression (4, 53). This difference in LRRK2 expression among cholinergic neurons correlated with differences in ciliogenesis: *Grm5* expressing neurons with higher LRRK2 expression were less ciliated than those expressing *Elavl2*.

Although overall gene expression changes were limited between WT and G2019S LRRK2 mouse dorsal striatum, oligodendrocytes, astrocytes, and microglia all showed a highly significant decrease in Ferritin heavy chain (Fth1) transcription in G2019S LRRK2 dorsal striatum. Oligodendrocytes are the major iron-containing cells in the brain and prior work knocking out one copy of ferritin heavy chain gene rendered cells much more sensitive to oxidative stress (54). It is not clear why expression of this gene is particularly changed in LRRK2 mutant cells, but decreased Ferritin heavy chain will surely have major consequences for the physiology of the respective cell classes. In addition, G2019S expressing, direct-, indirect, and eccentric-spiny neurons as well as parvalbumin interneurons showed increased expression of the CNTN5 neuronal adhesion protein. This parallels loss of dopaminergic process intensity in the dorsal striatum, monitored by both tyrosine hydroxylase and GDNF receptor immunofluorescence.

Analysis of postmortem human brain tissue showed low levels of astrocyte (~40%) and cholinergic neuron (~10%) ciliation in all samples from ~85-y-old patients, compared with ~70% ciliation in all striatal cells of 5-mo-old, WT mice. There were even fewer cilia in both LRRK2 pathway-related mutation carriers and idiopathic PD. Indeed, a rare patient with a deleterious truncation in PPM1H phosphatase showed the same low level of ciliation as a patient carrying the G2019S LRRK2 mutation. The lack of cilia will make these cells unable to sense Shh signals and consequently, unable to provide the same level of neuroprotection to their neighbors.

An important finding in this study was the observation that in 11 human PD brains analyzed (3 LRRK2 PD, 8 sporadic PD, and 1 PPM1H truncation), we detected a highly concordant and significant, two-fold loss of cholinergic neurons relative to four control samples. These findings are consistent with prior reports of loss of cholinergic markers in PD patients or animal models (reviewed in ref. 55). Loss of cholinergic interneurons is consistent with the loss of GDNF transcription that we detect in mouse and consistent with loss of neuroprotection for striatal dopamine neurons.

A remaining puzzle is why astrocyte and interneuron cilia are more sensitive to pathogenic LRRK2 expression than the predominant medium spiny neurons of the dorsal striatum in both mice and humans. Sensitivity between distinct cell type classes is not due to LRRK2 expression as our snRNAseq data showed that at least at the RNA level, LRRK2 is much more highly expressed in medium spiny neurons than in cholinergic neurons, and the counteracting PPM1H phosphatase is more highly expressed in the cells most vulnerable to LRRK2 action. Careful analyses with fully validated rabbit monoclonal antibodies have indicated that LRRK2 RNA and protein expression are concordant across the mouse brain (56). Future work will continue to explore the basis for the cell type specificity of LRRK2 citation inhibition.

In summary, our work reveals that specific cell types in the striatum of both mice and humans lose primary cilia due to the action of pathogenic LRRK2 kinase. Loss of cilia is seen in both LRRK2-pathway mutant carriers and in idiopathic PD, and the cause of cilia loss in idiopathic disease warrants further study. We show that loss of cilia leads to decreased GDNF production by cholinergic neurons that is needed for dopamine neuron viability. We see also loss of cholinergic neurons and dopaminergic processes, with upregulation of the neural adhesion protein, CNTN5. These data highlight the importance of neuroprotective pathways for the function of the nigrostriatal circuit.

In addition to neurons, LRRK2 G2019S astrocytes, oligodendrocytes, and microglia all showed a highly significant decrease in the ferritin heavy chain, making these important cells more vulnerable to oxidative stress. Future studies using cell-type-specific mutant LRRK2 expression will teach us much about the relative contribution of each of these cell types to PD.

## Materials and Methods

Key resources are listed in *SI Appendix*, Table S3.

### Single Nuclei RNAseq.

Isolation of nuclei from adult mice was carried out as described [Allen Institute for Brain Sciences, 2021 (10.17504/protocols.io.bq7emzje)]. Six-month-old female mice (three WT and three G2019S KI LRRK2) were transcardially perfused with 1× PBS. Brains were subsequently removed, dorsal striatal tissue was dissected in ice-cold PBS, placed in 1.5 mL microfuge tubes, and quickly frozen in a slurry consisting of 200 proof ethanol and dry ice. Tubes were stored at −80 °C before further processing.

For nuclei isolation, 1 mL of cold homogenization buffer (nuclei isolation media containing 0.1M DTT, 1× protease inhibitor cocktail (Sigma), 0.2 U/µL of RNAsein plus and 0.1% Triton X-100) was added to each tube, and once thawed, the tissues were transferred to dounce homogenizers as described (10.17504/protocols.io.bq7emzje). Each tissue sample was dounced 10 times with a loose pestle then 10 times with a tight pestle (20 strokes total). The resulting homogenates were filtered through 30-µm strainers to remove large debris and transferred to 15-mL conical tubes. Tubes were spun at 900×*g* for 10 min at 4 °C. Supernatants were removed, leaving only ~50 µL of solution. Samples were resuspended in 450 µL blocking buffer (1× PBS containing 0.8% BSA and 0.2 U/µL RNAsein). Then, 10 µL of each sample was combined with 10 µL of 0.4% Trypan Blue to assess nuclei quality and sample yield. Concentrations were then adjusted to 1,000 nuclei/µL with blocking buffer. Sequencing using 10× Chromium Single Cell 3′, 5′, T and B cell V(D)J (V2), 10× scATACSeq, 10× 3′ WTA (V3), and feature barcoding was then performed by Stanford Genomics.

### Data Preprocessing.

Seurat [version 3, https://satijalab.org/seurat/; (57)] was used for single-cell analysis (https://doi.org/10.5281/zenodo.10470951). Quality control steps were performed to identify and remove cells that were potential outliers. This included removing potential multiplets using “DoubletFinder” [https://github.com/chris-mcginnis-ucsf/DoubletFinder (58)] and “cells” that displayed high mitochondrial gene expression (using the subset function to remove clusters with high expression of “MT-” genes). The data were then normalized and log-transformed (using the “LogNormalize” method), and counts were scaled. All files, including the scripts used for Seurat gene expression analyses are available on Dryad (https://doi.org/10.5061/dryad.pk0p2ngvp).

### Analysis.

Seurat (57) was used on the aligned dataset to identify cell clusters, and then t-SNE or uMAP was used to visualize similarities between cells. Next, cell types were assigned to these clusters based on the expression of predefined marker genes [using Dropviz.org (18) and literature searches; see *SI Appendix*, Table S1 of top hits used for cluster_IDs].

### Research Standards for Human and Animal Studies.

The LRRK2^G2019S/G2019S^ mice were obtained from Taconic (Lrrk2tm4.1Arte #13940; ref. 59) and kept in specific pathogen-free conditions at the University of Dundee (UK). All animal studies were ethically reviewed and carried out in accordance with Animals (Scientific Procedures) Act 1986 and regulations set by the University of Dundee and the U.K. Home Office. Animal studies and breeding were approved by the University of Dundee ethical committee and performed under a U.K. Home Office project license. Mice were housed at an ambient temperature (20 to 24 °C) and humidity (45 to 55%) and maintained on a 12 h light/12 h dark cycle, with free access to food (SDS RM No. three autoclavable) and water. Genotyping of mice was performed by PCR using genomic DNA isolated from ear biopsies. Primer 1 (5′-CTGCAGGCTACTAGATGGTCAAGGT-3′) and Primer 2 (5′-CTAGATAGGACCGAGTGTCGCAGAG-3′) were used to detect the WT and knock-in alleles (10.17504/protocols.io.5qpvo3xobv4o/v1). Homozygous LRRK2-G2019S and littermate WT controls (5 mo of age) were used for mouse experiments. The genotypes of the mice were confirmed by PCR on the day of experiment.

### Immunohistochemistry and Microscopy.

Immunohistochemistry of the human brain lenticular nucleus was performed as previously described (10.17504/protocols.io.j8nlkowj1v5r/v1). Briefly, free-floating tissues were incubated with 10 mM sodium citrate buffer pH 6.0 (preheated to 95° C) for 15 min at 95 °C to retrieve the antigens. Tissues were permeabilized with 0.1% Triton X-100 in PBS at RT for 1 h. Tissues were blocked with 2% FBS and 1% BSA in PBS for 2 h at RT and were then incubated overnight at 4 °C with primary antibodies. The following day, tissues were incubated with secondary antibodies at RT for 2 h. Donkey highly cross-absorbed H+L secondary antibodies conjugated to Alexa 488, Alexa 568, or Alexa 647 were used at a 1:2,000 dilution. Nuclei were stained with 0.1 µg/mL DAPI (Sigma). Tissues were incubated with freshly prepared 0.1% Sudan Black B in 70% ethanol for 20 min to reduce autofluorescence. Stained tissues were transferred to slides and overlayed with Fluoromount G and a glass coverslip. All antibody dilutions for tissue staining included 1% DMSO to help antibody penetration. All images were obtained using a Zeiss LSM 900 confocal microscope with a 63 × 1.4 oil immersion objective or 20×/0.8 objective. All image visualizations and analyses were performed using Fiji (60). A summary of the areas imaged for each sample is included in *SI Appendix*, Table S4.

Immunostaining of the mouse brain striatum was performed as previously described (10.17504/protocols.io.j8nlkowj1v5r/v1): Frozen slides were thawed at RT for 15 min and then gently washed (2X) with PBS for 5 min. Slides were incubated with 10 mM sodium citrate buffer pH 6.0 (preheated to 95° C) for 15 min at 95° C to retrieve the antigens. Sections were permeabilized with 0.1% Triton X-100 in PBS at RT for 15 min. Sections were blocked with 2% FBS and 1% BSA in PBS for 2 h at RT and were then incubated overnight at 4 °C with primary antibodies. The following day, sections were incubated with secondary antibodies at RT for 2 h. Donkey highly cross-absorbed H+L secondary antibodies conjugated to Alexa 488, Alexa 568, or Alexa 647 were used at a 1:2,000 dilution. Nuclei were stained with 0.1 µg/mL DAPI (Sigma). Stained tissues were overlayed with Fluoromount G and a glass coverslip. All antibody dilutions for tissue staining included 1% DMSO to help antibody penetration. All images were obtained using a Zeiss LSM 900 confocal microscope with a 63 × 1.4 oil immersion objective. All image visualizations and analyses were performed using Fiji (60) and CellProfiler (61). RNAscope fluorescence in situ hybridization was carried out according to the manufacturer as described (16).

In addition to above-mentioned methods, all other statistical analysis was carried out using GraphPad Prism version 9.3.1 for Macintosh, GraphPad Software, Boston, MA, www.graphpad.com.

## Supplementary Material

Appendix 01 (PDF)

Dataset S01 (XLSX)

## Data Availability

All primary data have been deposited in DRYAD (https://doi.org/10.5061/dryad.pk0p2ngvp) (62).
