# Supplementary material for: Loss of primary cilia and dopaminergic neuroprotection in pathogenic LRRK2-driven and idiopathic Parkinson’s disease
Source: Proc Natl Acad Sci U S A. 2024 Aug 1;121(32):e2402206121. doi: 10.1073/pnas.2402206121 (PMC11317616; doi:10.1073/pnas.2402206121)
Supplement: Supplementary file 1 — Appendix 01 (PDF) [file pnas.2402206121.sapp.pdf]

PNAS

Supporting Information for

Loss of primary cilia and dopaminergic neuroprotection in pathogenic LRRK2-driven and idiopathic Parkinson's disease

Shahzad S. Khan, Ebsy Jaimon, Yu-En Lin, Jonas Nikoloff, Francesca Tonelli, Dario R. Alessi, and Suzanne R. Pfeffer

Corresponding author: Suzanne R. Pfeffer
Email: pfeffer@stanford.edu

This PDF file includes:

Tables S1 to S5
Legend for Dataset S1

Other supporting materials for this manuscript include the following:

Dataset S1

Table S1. Markers used to identify cell type clusters

Cluster ID	Key Markers
**dSPN** Direct spiny neuron	Drd1, Lingo2
**iSPN** Indirect spiny neuron	Drd2, Adora2a
**Oligo** Oligodendrocyte	Stl18, Pex5l
**Astrocyte**	Gpc5, Slc1a2
**Microglia**	Cx3cr1, Inpp5d
**NPC** Neuronal precursor cell	Sox4 Dlx1
**OPC** Oligodendrocyte precursor	Tnr, Pdgfra
**eSPN** Eccentric spiny neuron	Otof, Adarb2
**Pvalb_IN** Parvalbumin interneuron	Kit, Nxph1, Ilrap2
**Endothelial**	Flt1, Slcola4
**Ependymal**	Dnah12, Tmem112
**Mural**	Atp13a5
**Sst_IN** Somatostatin interneuron	Nos1
**Chat_IN** Cholinergic interneuron	Cpne4, Clstn2
**Fibroblast**	Xfp804b

Table S2. Top ten gene lists for astrocytes and cholinergic neurons.

A. Top 10 gene list for each astrocyte subcluster

Cluster ID	Gene List
Aldh1a1	Aldh1a1, Fry, C730002L08Rik, Unc13c, Cntn1, Luzp2, Trpm3, Egfem1, Gpr158, Otud7a
Crym	Crym, Pde10a, Robo1, Zswim6, Gabbr2, Pde7b, Hdac4, Gm12239, Ncam2, Hes5
Nrg1	Nrg1, Adamts18, Csmd1, Slit2, Ldb2, Nkain2, Meg3, Adgrv1, Tox3, Prr16
Gfap	Igfbp5, Thbs4, Kcnj3, Prkca, Clu, Garnl3, Gfap, Apoe, Gja1, Aqp4

B. Top 10 gene list for each cholinergic interneuron subcluster

Cluster ID	Gene List
Elavl2	Elavl2, Luzp2, Inpp4b, Tacr1, Asic2, Slc7a14, Clstn2, Cpne4, Trpc3, Rgs6
Grm5	A330015K06Rik, Grm5, Ptprd, Kcnq5, Pde10a, Pde7b, Rarb, Meis2, Cntn5, Plcb1

Table S3. Patient information related to human samples analyzed

Case ID	gender	Unified Lewy Body Stage*	Summary	Age at diagnosis	Age at death
01-31	male	0. No Lewy bodies	Control		81
01-46	female	0. No Lewy bodies	Control		≥90
02-27	male	0. No Lewy bodies	Control		86
03-63	female	0. No Lewy bodies	Control		83
100	female	0. No Lewy bodies	Control		70
249	female	0. No Lewy bodies	Control		59
418	male	0. No Lewy bodies	Control		74
10-28	male	IV. Neocortical	Parkinson's disease	55	75
10-37	female	III. Brainstem/Limbic	Parkinson's disease; Neurofibrillary tangles, **LRRK2 G2019S**	64	84
14-03	male	IV. Neocortical	Parkinson's disease; Alzheimer's disease; Vascular dementia; **p.E309X PPM1H**	85	≥90
01-39	male	III. Brainstem/Limbic	Parkinson's disease; Alzheimer's disease; **LRRK2 G2019S**	74	85
13-60	male	0. No Lewy bodies	Alzheimer's disease; Parkinsonism; **LRRK2 G2019S** (homozygous)	85	89
16-23	female	IV. Neocortical	Parkinson's disease	61	78
18-72	female	IV. Neocortical	Parkinson's disease	67	81
12-03	male	IV. Neocortical	Parkinson's disease	65	87
12-56	female	III. Brainstem/Limbic	Parkinson's disease	57	70
10-27	female	IV. Neocortical	Parkinson's disease	69	85
19-73	male	IV. Neocortical	Parkinson's disease	70	84
21-81	male	IV. Neocortical	Parkinson's disease; Alzheimer's disease		82
872	female	PD related Braak V	Parkinson's disease	N/A	82
1047	female	PD related Braak IV	Parkinson's disease	N/A	83
643	male	PD related Braak IV	Parkinson's disease	N/A	78

*Unified LB stage is the synuclein/Lewy body stage defined using the Unified Staging System for Lewy Body Disorders (Beach TG et al Acta Neuropathol.117:613-634, 2009).

Table S4. Key Resources used in this study

Reagent type (species) or resource	Designation	Source or reference	Identifiers	Additional information
Genetic reagent(Mus musculus)	Constitutive KI Lrrk2tm4.1Arte	Taconic	#13940, RRID:IMSR_TAC:13940	C57BL/6; G2019S KI
Genetic reagent(Mus musculus)	Ppm1h-/- mouse	Taconic	#TF3142	C57BL/6 Background
Antibody	anti-Choline Acetyltransferase (goat polyclonal)	Millipore	AB144P-1ML (RRID:AB_2079751)	(1:200)
Antibody	anti-Adenylate cyclase III (mouse monoclonal)	Santa Cruz	SC-518057 (RRID:AB_3073967)	(1:100)
Antibody	anti-DARPP-32 (rabbit monoclonal)	Cell Signaling Technology	#2306S (RRID:AB_823479)	(1:400)
Antibody	anti-GFAP (chicken polyclonal)	EnCOR	CPCA-GFAP (RRID:AB_2109953)	(1:2000)
Antibody	anti-Arl13B (mouse monoclonal)	Neuromab	N295B/66 (RRID:AB_2877361)	(1:500)
Antibody	anti-GFR alpha-1/GDNF R alpha-1 (goat polyclonal)	R&D Systems	AF560 (RRID:AB_2110307)	(1:500)
Antibody	anti-Tyrosine hydroxylase (sheep polyclonal)	Novus Biologicals	NB300-110 (RRID:AB_10002491)	(1:500)
Antibody	anti-Cntn5 (rabbit polyclonal)	Novus Biologicals	NBP1-83242 (RRID:AB_11019867)	(1:50)
Antibody	anti-NeuN(chicken polyclonal)	Millipore	ABN91 (RRID:AB_11205760)	(1:1000)
Antibody	anti-DRD2(mouse monoclonal)	NeuroMab	N186/29 (RRID:AB_11000721)	(1:250)
Antibody	H+L Donkey anti-mouse Alexa 488	Life Technologies	A32766 (RRID:AB_2762823)	(1:2000)
Antibody	H+L Donkey anti-Rabbit Alexa 568	Life Technologies	A10042 (RRID:AB_2534017)	(1:2000)
Antibody	H+L Donkey anti-goat Alexa 488	Life Technologies	A11055 (RRID:AB_2534102)	(1:2000)
Antibody	H+L Donkey anti-mouse Alexa 647	Life Technologies	A31571 (RRID:AB_162542)	(1:2000)
Antibody	H+L Donkey anti-chicken Alexa 488	Jackson ImmunoResear ch	#703-545-155 (RRID:AB_2340375)	(1:2000)
Antibody	H+L Donkey anti-sheep Alexa 488	Life Technologies	A-11015 (RRID:AB_2534082)	(1:2000)
Antibody	H+L Donkey anti-goat Alexa 568	Life Technologies	A-11057 (RRID:AB_2534104)	(1:2000)
Reagent	Sudan Black B	Chem-Impex International	#01307	
Commercial assay or kit	RNAscopeMultiplexFluorescentReagent Kit v2	Advanced Cell Diagnostics	#323100	
Commercial assay or kit	RNAscope Probe- Mm-Lrrk2	Advanced Cell Diagnostics	#421551	(1:20)
Commercial assay or kit	RNAscope Probe-Mm-Grm5-C3	Advanced Cell Diagnostics	#423631-C3	
Commercial assay or kit	OPAL 570 REAGENT PACK	Akoya Biosciences	FP1488001KT	

Commercial assay or kit	OPAL 690 REAGENT PACK	Akoya Biosciences	FP1497001KT	
Software, Algorithm	FIJI	PMID:29187165<br>Version 2.9.0	RRID:SCR_002285	
Software, Algorithm	CellProfiler 4.2.6	PMID:29969450	RRID:SCR_007358	
Software, Algorithm	Prism	Prism Version 9.3.1 (350)	RRID:SCR_002798	
Software, Algorithm	RStudio	https://posit.co/<br>Version 2023.09.1	RRID:SCR_000432	
Software, Algorithm	Seurat	PMID:29608179<br>Version 4.4.0	RRID:SCR_016341	
Software, Algorithm	ImageJ	https://imagej.net/<br>Version 1.54h	ImageJ RRID:SCR_003070	
Software, Algorithm	DoubletFinder	PMID:30954475<br>Version 2020.03.31	RRID:SCR_018771	

Table S5. Summary of the areas analyzed for each Lenticular nucleus tissue sample

Brain ID	Tiles Analyzed	Area Analyzed (mm^2)	ChAT+ neurons
01-31 (control)	1017	87.60	309
01-46 (control)	1082	91.99	444
02-27 (control)	1078	92.63	413
03-63 (control)	1041	82.67	362
01-39 (G2019S)	645	55.75	90
10-37 (G2019S)	117	10.59	26
13-60 (G2019S)	637	54.80	99
10-28 (sporadic PD)	940	80.36	143
21-81 (sporadic PD)	103	9.52	28
12-56 (sporadic PD)	101	9.36	21
10-27 (sporadic PD)	144	12.35	25
19-73 (sporadic PD)	115	10.09	26
12-03 (sporadic PD)	144	12.35	26
14-03 (PPM1H)	513	44.28	87
16-23 (sporadic PD)	496	42.58	95
18-72 (sporadic PD)	570	46.36	72

Dataset S1.

This Excel file reports the differentially expressed genes from comparisons of G2019S LRRK2 knockin (KI) and wildtype nuclei from the mouse dorsal striatum in our single nucleus RNA sequencing dataset. This file is also available on Dryad (https://doi.org/10.5061/dryad.pk0p2ngvp). Individual sheets in the file report the results for a given cell type cluster/subcluster as indicated. Differentially expressed genes were identified using the Seurat "FindMarker()" command in RStudio. The columns list gene names (**Names**), p values (**p_val**), log2 fold changes (**log2FoldChange**), percentage of nuclei from G2019S KI mice expressing the listed gene (**pct.1**), percentage of nuclei from WT mice expressing the listed gene (**pct.2**), adjusted p values (**pad**j), number of reads obtained from G2019S KI mice for the listed gene (**Counts_GS**), and the number of reads obtained from WT mice for the listed gene (**Counts_WT**).
